# Interleukin-27 Gene Delivery Targeting IL-6Rα-Expressing Cells as a Stress Response Therapy

**DOI:** 10.3390/ijms21031108

**Published:** 2020-02-07

**Authors:** Manoel Figueiredo Neto, Shengzhi Liu, Janelle Wes Salameh, Hiroki Yokota, Marxa Leão Figueiredo

**Affiliations:** 1 Department of Basic Medical Sciences and Interdisciplinary Biomedical Sciences Program, Purdue University, 625 Harrison Street, LYNN 2177, West Lafayette, IN 47907, USA; netobrgo@hotmail.com (M.F.N.); wsalameh@purdue.edu (J.W.S.); 2 Department of Biomedical Engineering, Indiana University Purdue University Indianapolis, Indianapolis, IN 46202, USA; liu441@iupui.edu (S.L.); hyokota@iupui.edu (H.Y.); 3 Purdue Center for Cancer Research and Purdue Institute for Drug Discovery, Purdue University, West Lafayette, IN 47907, USA

**Keywords:** interleukin-27, peptide targeting, gene delivery, prostate cancer, RNAseq, eIF2α

## Abstract

Interleukin-27 (IL-27) has shown promise in halting tumor growth and mediating tumor regression in several models, including prostate cancer. We describe our findings on the effects of IL-27 on the gene expression changes of TC2R prostate adenocarcinoma cells. We utilized RNAseq to assess profile differences between empty vector control, vector delivering IL-27 modified at its C-terminus with a non-specific peptide, and IL-27 modified at the C-terminus with a peptide targeting the IL-6-Rα. The targeted IL-27 had higher bioactivity and activity in vivo in a recent study by our group, but the mechanisms underlying this effect had not been characterized in detail at the gene expression level on tumor cells. In the present work, we sought to examine potential mechanisms for targeted IL-27 enhanced activity directly on tumor cells. The targeted IL-27 appeared to modulate several changes that would be consistent with an anti-tumor effect, including upregulation in the Interferon (IFN) and Interferon regulatory factor (IRF), oxidative phosphorylation, Janus kinase/Signal transducers and activators of transcription (JAK/STAT), and eukaryotic initiation factor 2 (EIF2) signaling. Of these signaling changes predicted by ingenuity pathway analyses (IPA), the novel form also with the highest significance (-log(Benjamini–Hochberg (B-H)) *p*-value) was the EIF2 signaling upregulation. We validated this predicted change by assaying for eukaryotic initiation factor 2 alpha (eIF2α), or phosphorylated eIF2α (p-eIF2α), and caspase-3 levels. We detected an increase in the phosphorylated form of eIF2α and in the cleaved caspase-3 fraction, indicating that the EIF2 signaling pathway was upregulated in these prostate tumor cells following targeted IL-27 gene delivery. This approach of targeting cytokines to enhance their activity against cancer cells is a novel approach to help augment IL-27′s bioactivity and efficacy against prostate tumors and could be extended to other conditions where it could help interfere with the EIF2α pathway and promote caspase-3 activation.

## 1. Introduction

The cytokine interleukin (IL)-27 is a heterodimeric cytokine composed of subunits IL-27p28 and EBI3 (Epstein–Barr virus-induced gene 3), which are related to the IL-12 subunits p35 and p40, respectively. IL-27 is a therapeutic under development by our group for arthritis [1] and malignant tumors [2,3,4], on the basis of its multifunctional (immune stimulatory, anti-angiogenic, pro-osteogenic) activity. IL-27 has key functions in bone repair, helping prevent osteoclast formation and promoting osteoblast differentiation [2,3], and these features can be used to treat various forms of arthritis as well as bone-metastatic tumors. The receptor for IL-27, a heterodimer composed of IL-27 receptor alpha (IL-27RA) and glycoprotein 130 (gp130) subunits, is highly expressed in lymphoid organs, bone, normal and tumor epithelial cells [5,6], melanoma [7], and leukemia [8]. IL-27 signaling induces T-bet, Interferon gamma (IFNγ), and IL12-Rβ2 expression, promoting initiation of Th1 differentiation [9,10]. Either systemic [11] or intratumoral [2] IL-27 treatments eliminate tumors without toxicity, and mostly the anti-tumor mechanisms have been shown to be indirect, including induction of cytotoxic T lymphocyte responses or inhibition of angiogenesis through induction of chemokine (C-X-C motif) ligand (CXCL)9-10 [11].

Our previous studies using gene delivery of IL-27 significantly reduced the rate of tumor growth and normalized in vivo bone density [4], potentially through recruitment of immune effectors to tumors; however, mechanisms by which IL-27 can promote direct effects on tumor cells remains incompletely characterized. Our group aimed to enhance the efficacy of IL-27 on tumor cells by modifying the cytokine at the C-terminus with a targeting peptide or ligand-targeted approach [12]. We hypothesized that targeting the cytokine to tumor tissue by utilizing peptides that could bind receptors upregulated in tumor cells, such as the interleukin-6 receptor (IL-6Rα), could help augment IL-27 bioactivity. The IL-6Rα (and Signal transducers and activators of transcription 3 (STAT3) oncogenic pathway) expression in aggressive, castration-resistant prostate cancer has recently been assessed in patients, making this receptor and pathway of high interest for targeting therapeutic interventions [13]. For achieving targeting of the IL-6Rα, we selected a candidate heptapeptide from the literature, Leu-Ser-Leu-Ile-Thr-Arg-Leu (LSLITRL or pepL), which was first identified from a 7 mer random cyclic phage display screen targeting this receptor [14]. This pepL inhibited IL-6 binding to IL-6Rα in a dose-dependent manner and could bind to the plasma membrane of IL-6Rα-expressing cell lines. We utilized this targeted IL-27 and reported that it displayed higher bioactivity and activity in vivo relative to control IL-27 or empty vector in a recent study [12]. One limitation of our current knowledge has been, however, that the mechanisms underlying the higher bioactivity of targeted IL-27pepL had not been characterized in detail at the gene expression level and directly on tumor cells.

In the present work, we sought to examine potential mechanisms for targeted IL-27-enhanced activity directly on tumor cells utilizing RNA-sequencing (RNAseq). The targeted IL-27 appeared to modulate several changes that would be consistent with an anti-tumor effect, including upregulation in the IFN and Interferon regulatory factor (IRF) signaling, oxidative phosphorylation, JAK/STAT signaling, and eukaryotic initiation factor 2 (EIF2) signaling. Of these signaling changes predicted by ingenuity pathway analyses (IPA), the one with highest significance (−log(Benjamini–Hochberg (B-H)) *p*-value) was the eIF2 signaling upregulation. We validated this signaling change by assaying for eukaryotic initiation factor 2 alpha (eIF2α), phosphorylated eIF2α (p-eIF2α), and caspase-3 levels. We detected an increase in the phosphorylated form of eIF2α and in the cleaved caspase-3 fraction, indicating that the eIF2α signaling pathway was upregulated in these prostate tumor cells following targeted IL-27 gene delivery, leading to apoptosis induction. This approach of targeting cytokines to enhance their activity against cancer cells is a novel approach to help augment IL-27′s bioactivity and efficacy against prostate tumors, and could be extended to other conditions where it could help interfere with the eIF2α pathway and promote caspase-3 activation.

Prostate cancer is the second most prevalent cancer in men worldwide. Despite the advances in understanding the molecular processes driving the onset and progression of this disease, as well as the continued implementation of screening programs, this cancer still remains a significant cause of morbidity and mortality. It is only recently that defects of the cellular translation machinery have begun to gain attention as an important cause of cancer development in different tissues, including the prostate. In particular, the initiation step of translation has been established as having a key role in tumorigenesis. Our results suggest that the targeted form of IL-27 may be utilized as a strategy impacting cell translation initiation through modulating the eIF2α pathway (stress to the endoplasmic reticulum (ER) leading to apoptosis) as a novel approach for treating prostate cancer.

## 2. Results

### 2.1. Different Global Gene Expression Analyses Following RNAseq Data Collection Showed Commonalities and Differences for These Gene Therapy-Based IL-27 Therapeutic Candidates

**Figure 1:**
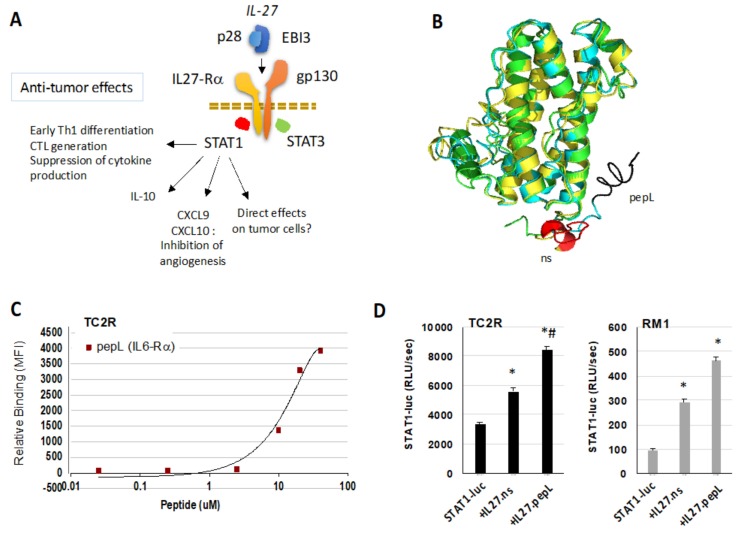
IL-27 cytokine and peptide characterization. (**A**) Signaling switches towards Signal transducers and activators of transcription 1 (STAT1) in IL-27-stimulated cells, promoting anti-tumor effects in the tumor microenvironment. The effects of IL-27 directly on tumor cells are not as well-characterized as on immune cells. (**B**) The IL-27p28 subunit was modeled with one linker and peptides (nonspecific (ns), or LSLITRL (PepL)) appended at the C-terminus via genetic modification. These modified p28s showed a high degree of homology. (**C**) TRAMPC2-Ras (TC2R) cell binding assay for a concentration range of PepL conjugated to fluorescein isothiocyanate (FITC) and assessed by flow cytometry; MFI, mean fluorescence intensity. (**D**) STAT1-luciferase (luc) reporter assays for TC2R and Ras/myc (RM1) cell lines, showing that cotransfection with an IL-27ns plasmid enhanced STAT1 activity (* *p* < 0.05 relative to baseline) and cotransfection with a IL-27pepL plasmid enhanced STAT1 activity further (# *p* < 0.05 relative to IL-27ns).

Prostate cancer cells TRAMPC2-Ras (TC2R) were transfected with control empty vector (plasmid DNA pcDNA3.1), or the same backbone vector containing control IL-27 with a non-specific peptide, or the targeted form of IL-27 (IL-27pepL, targeted to the IL-6Rα), as described in the Materials and Methods section. IL-27 was selected for its anti-tumor activity mechanisms, as reported in the literature and summarized in Figure 1a. Computer modeling indicated that the targeting peptides (nonspecific (ns), or targeted to IL-6Rα (pepL)) did not alter the overall structure of the cytokine (Figure 1b). The binding of a fluorescein isothiocyanate (FITC)-labeled pepL to TC2R cells was examined by flow cytometry and the EC_50_ estimated at ≈10 µM (Figure 1c). Additionally, we examined the efficacy of the IL-27 cytokines modified at the C-terminus in a STAT1-luc reporter assay, where co-transfection of IL-27ns vector augmented the STAT1 activity in both TC2R and Ras/Myc murine prostate adenocarcinoma cells (RM1) cell lines (Figure 1d, *p* < 0.05). Co-transfection of the IL-27pepL vector further augmented the STAT1 activity in both cell lines, but this difference was only significant for TC2R cells (*p* < 0.05).

**Figure 2:**
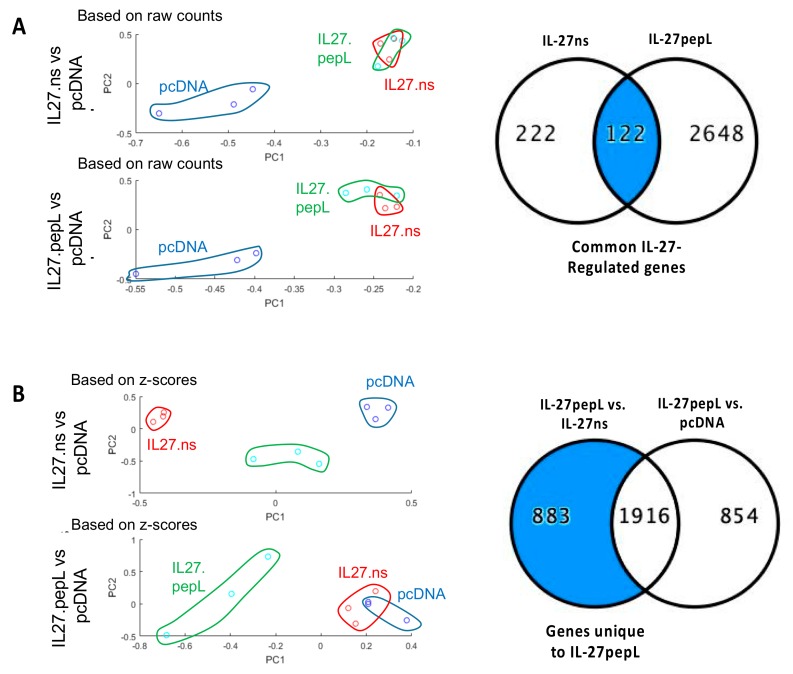
Different global gene expression analyses following RNA-sequencing (RNAseq) data collection showed commonalities and differences for these gene therapy-based IL-27 therapeutic candidates. Prostate cancer cells TRAMPC2-Ras (TC2R) were transfected with control empty vector (plasmid DNA pcDNA3.1), the same backbone vector containing control IL-27 with a non-specific peptide, or the targeted form of IL-27 (IL-27pepL, targeted to the IL-6Ra). (**A**) With principal component analyses (PCA) using raw counts following RNAseq, we observed a clustering of the IL-27 therapeutics in a separate group relative to pcDNA control, and the changes correlated with the ingenuity pathway analyses (IPA, right Venn diagram). (**B**) With PCA analyses based on z-scores, we observed a separation between the IL-27pepL and the other groups. The IL-27pepL therapy clustered separately and with IPA (Venn diagram), with many genes distinctly upregulated in the IL-27pepL group.

We performed two analyses to examine the impact of IL-27 therapeutics as compared to the empty vector control, as well as one-another. The first analyses utilized principal component analyses (PCA) and raw counts following RNAseq data collection. With PCA analyses, we observed a distinct clustering of the IL-27 therapeutics in a separate group relative to pcDNA control (Figure 2a), and the changes correlated with the ingenuity pathway analyses (IPA) performed (Figure 2a, Venn diagram). There were 122 genes commonly regulated between IL-27ns and IL-27pepL therapeutics. The second analyses utilized z-scores to examine whether the IL-27pepL therapeutic had a distinct pattern of gene expression as assessed by RNA seq relative to IL-27ns or empty vector control (Figure 2b). The IL-27pepL therapy clustered separately and with IPA (Figure 2b, Venn diagram), and there were 883 genes distinctly upregulated in the IL-27pepL group.

### 2.2. Ingenuity Pathway Analyses (IPA) Reported Specific Upstream Regulators and Canonical Pathways Differentially Modulated by IL-27pepL

**Figure 3:**
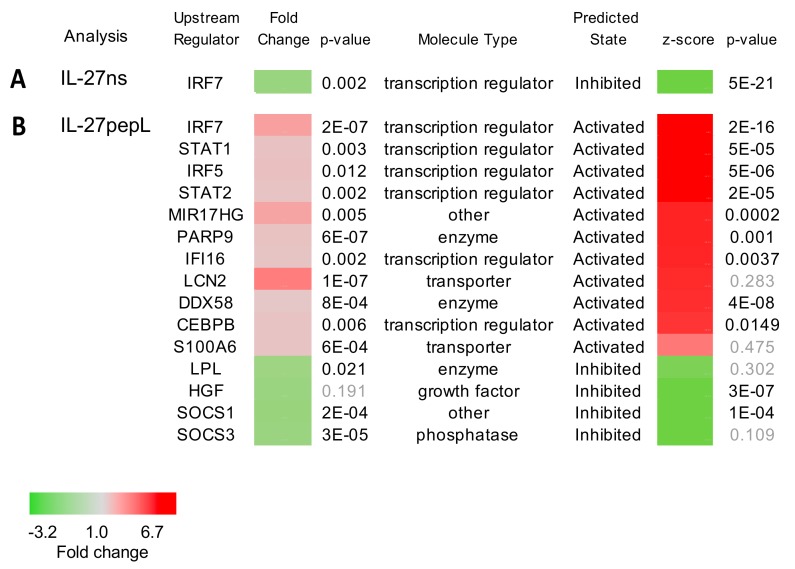
Upstream regulators predicted by IPA analysis of the dataset differentially expressed for (**A**) IL-27ns and (**B**) IL-27pepL, each relative to pcDNA empty vector control. Shown are the upstream regulators actual gene expression fold change as assessed by RNAseq, and the *p*-value. Also shown are molecule types, and the IPA-predicted state (activated or inhibited), the z-score of the predicted state, and its *p*-value. Grey: insignificant *p*-values; color bar: fold change.

Our first analysis utilized the “upstream regulators” modality of IPA, and it predicted regulators involved in the IL-27pepL therapy relative to the IL-27ns or empty vector control. These included some themes, with downregulation of IRF7 in the IL-27ns group (Figure 3a), and upregulation and predicted activation of several regulators including IRF7/5 and STAT1/2, which are related to known IL-27 signaling pathways (Figure 3b). Novel transcription regulators upregulated and predicted to be activated in the IL-27pepL group included interferon-inducible protein 16 (IFI16) and CCAAT/enhancer binding protein beta (CEBPB). Other regulators upregulated included microRNA 17 host gene (MIR17HG), protein mono-ADP-ribosyltransferase 9 (PARP9), Lipocalin 2 (LCN2), DExD/H-box helicase 58 (DDX58), and S100 calcium binding protein A6 (S100A6) (Figure 2b). Regulators that were downregulated and predicted to be inhibited included lipoprotein lipase (LPL), suppressor of cytokine signaling 1 and 3 (SOCS1/3), and hepatocyte growth factor (HGF). On the basis of these regulators predicted to potentially mediate the effects of the IL-27pepL therapy, we sought to determine individual genes or pathways downstream of these regulators and examine any novel trends.

**Figure 4:**
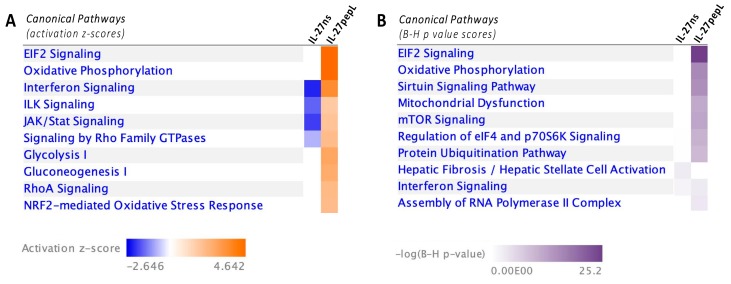
IPA canonical pathway analyses. Analyses of the dataset compared the control IL-27ns to the IL-27pepL therapeutic (both relative to pcDNA control). (**A**) The top five changes by activation z-score ranking (A) were eukaryotic initiation factor 2 (EIF2) signaling, oxidative phosphorylation, interferon signaling, integrin linked kinase (ILK) signaling, and Janus kinase (JAK)/STAT signaling. Color bar: activation z-score. (**B**) When the Benjamini–Hochberg (B-H) *p*-value score analysis was used, we observed the highest significant changes to include EIF2, oxidative phosphorylation, and mitochondrial dysfunction among the top five. Color bar: B-H *p*-value scores.

Our second analyses utilized different IPA comparative analyses to build pathways impacted by the gene expression changes observed by the different IL-27ns and IL-27pepL therapies relative to control empty vector. The first analysis utilized the “canonical pathways” modality of IPA using the activation z-scores data, and it predicted the top pathways differentially upregulated by IL-27pepL. Among these canonical pathways, EIF2, oxidative phosphorylation, interferon signaling, integrin linked kinase (ILK) signaling, and JAK/STAT signaling were the top five most upregulated in tumor cells that received the vector expressing IL-27pepL (Figure 4a). The second analysis utilized the “canonical pathways” modality of IPA but utilized the B-H *p*-value scores to represent the most significant changes related to expression of IL-27pepL in prostate cancer cells (Figure 4b).

### 2.3. Expanded Analyses of the Top Five Canonical Pathways Differentially Activated by Targeted IL-27 Relative to Controls

**Figure 5:**
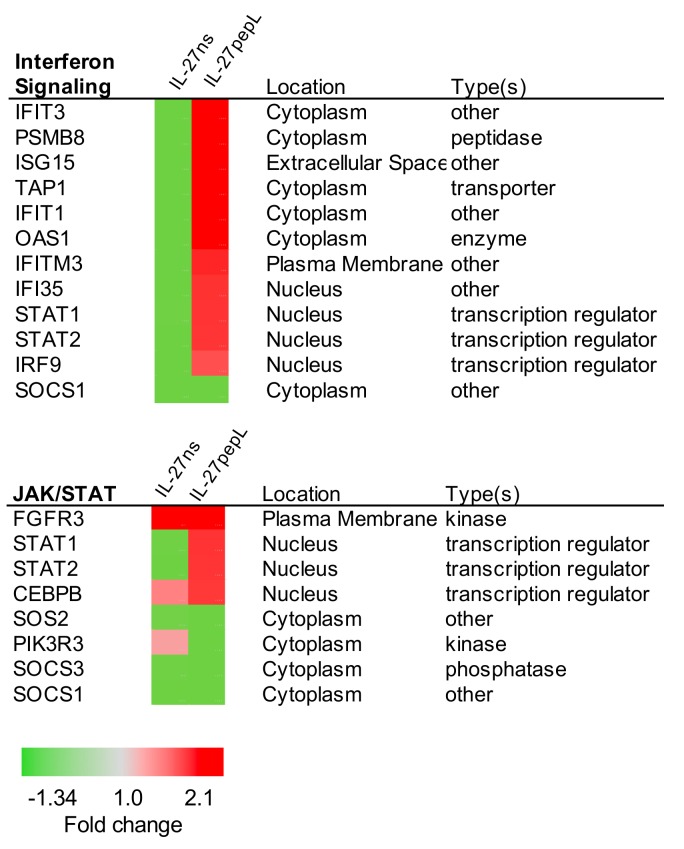
Top canonical pathways and regulators that differed between IL-27pepL and controls. This figure shows the signaling pathways previously reported as known for IL-27-regulated genes following IPA analysis including interferon signaling (top) and JAK/STAT signaling (bottom) and their differentially expressed gene list. Color bar: fold change.

We focused our analyses on the top five canonical pathways differentially activated by the targeted IL-27pepL therapeutic relative to IL-27ns or empty vector control. These pathways revealed two known pathways that relate closely to IL-27 cytokine signaling, interferon signaling and JAK/STAT, and they showed some overlap, in particular with upregulation of STAT1/2 transcriptional factors and downregulation of SOCS1. The specific gene changes and fold-change comparison between the IL-27ns and IL-27pepL therapies using ingenuity pathway analysis (IPA) are shown in Figure 5. Another analysis used *Enrichr*, using the ENCODE Transcription Factors (TF) ChIPseq dataset, an analysis that showed the top regulators of the gene list to cluster as enriched in STAT1/2, IRF1, and EP300 binding sites, and seemed to converge on either IRF1 or CEBPB as central network nodes (data not shown).

**Figure 6:**
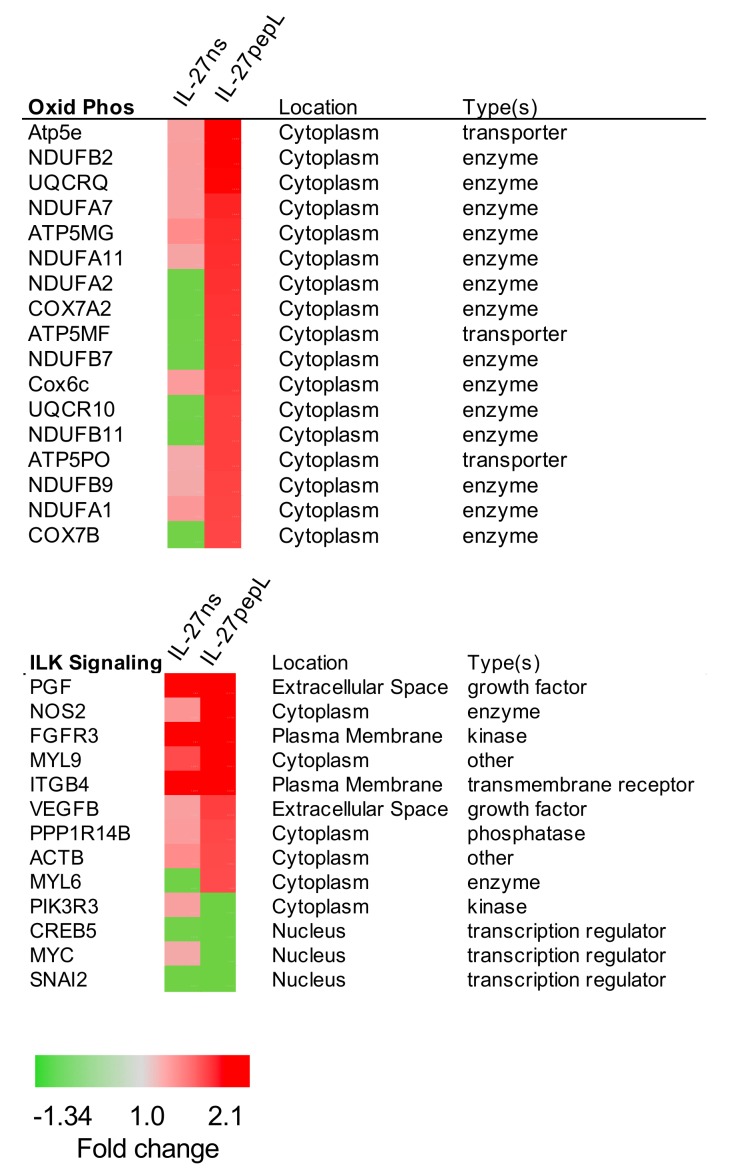
Top canonical pathways that differed between IL-27pepL and controls. This figure shows the two novel signaling pathways for signaling promoted by IL-27pepL, including oxidative phosphorylation (top) and ILK signaling (bottom). Color bar: fold change.

Interestingly, there were three pathways that were not previously reported as being modulated by IL-27 signaling. These included EIF2, oxidative phosphorylation, and ILK signaling. The specific gene changes and fold-change comparison between the IL-27ns and IL-27pepL therapies are shown in Figure 6 for the oxidative phosphorylation and ILK pathways. The oxidative phosphorylation pathway showed 17 gene expression changes, and the ILK signaling pathway showed 9 genes upregulated and 3 genes downregulated in expression.

**Figure 7:**
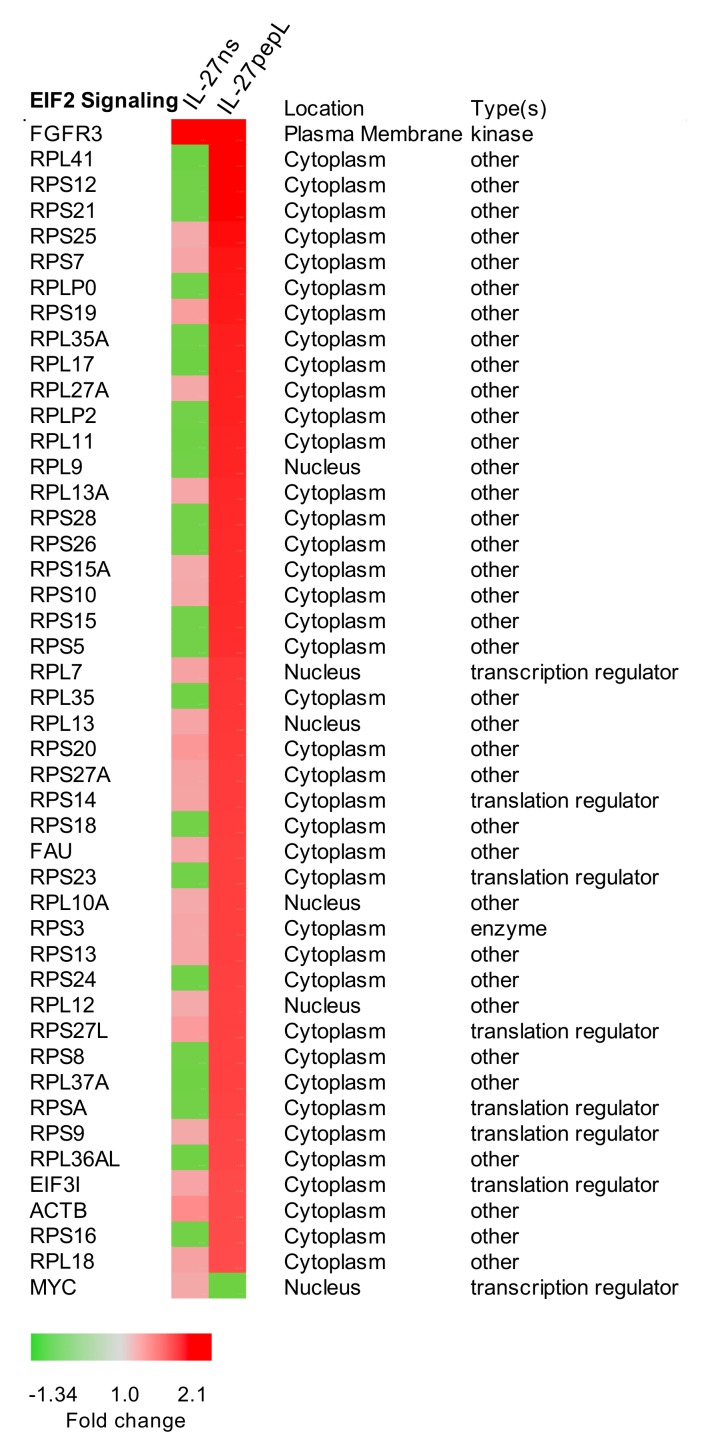
Top canonical pathways that differed between IL-27pepL and controls. This figure shows the most expressive novel signaling pathways for signaling promoted by IL-27pepL, EIF2α signaling. Color bar: fold change.

The EIF2 signaling pathway relates to translation initiation control and was the most expressively altered following IL-27pepL therapy, with 45 genes upregulated and 1 downregulated in this group. The specific gene changes and fold-change comparison between the IL-27ns and IL-27pepL therapies for the EIF2 pathway are shown in Figure 7.

### 2.4. The Top Novel IL-27pepL-activated EIF2 Pathway Could Be Validated at the Protein Level

**Figure 8:**
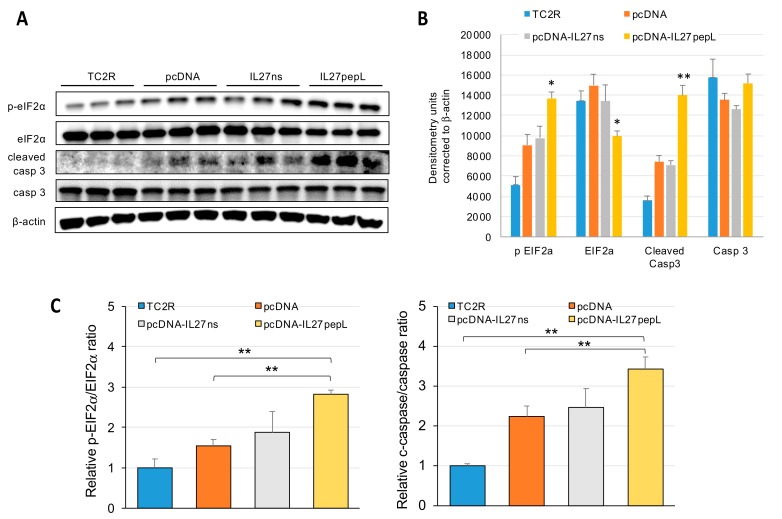
Western blots assessed protein and functional status relating to the EIF2 pathway. The eIF2 pathway protein alterations, such as levels of eIF2α and its phosphorylation status (**A**), as well as functional validation of the eIF2 pathway by levels of caspase-3 and cleaved caspase-3 quantification, were assayed by Western blot. (**B**) Quantification by densitometry of Western blots corrected to α-actin housekeeping gene control. (**C**) Determination of p-eIF2α/eIF2α and cleaved caspase-3 (cleaved Casp3)/caspase-3 (Casp3) ratios; * *p* < 0.05, ** *p* < 0.01. C, control TC2R cells without any plasmid; pcDNA, cells that received empty pcDNA3.1 vector, i.e., lacking insert.

To validate the top novel pathway, EIF2 signaling, predicted to be activated by IL-27pepL, we examined the levels of total and phosphorylated eIF2α by Western blots. The total levels of eIF2α were significantly reduced, whereas the levels of phosphorylated eIF2α were augmented significantly in IL-27pepL-treated TC2R cells (Figure 8a). The eIF2α changes corresponded with an increase in activated or cleaved caspase-3, following densitometric quantitation of the Western blots (Figure 8b). These changes may relate to endoplasmic reticulum (ER) stress responses, typically correlating with increases in eIF2α phosphorylation levels and accompanying caspase-3 activation and apoptosis of cancer cells. Analysis of the ratios of relative p-eIF2α to eIF2α showed a significant increase in p-eIF2α and cleaved caspase-3 (Casp3) relative to controls for the IL27pepL-treated group only (*p* < 0.01) (Figure 8c).

## 3. Discussion

In this work we sought to examine potential mechanisms for the enhanced activity of targeted IL-27 (IL-27pepL) directly on tumor cells following gene delivery and as compared to controls, utilizing RNAseq. We chose RNAseq over microarray for its many advantages over microarrays, including its ability to detect novel transcripts, as well as detect a higher percentage of differentially expressed genes, especially low abundance transcripts [15,16]. Additionally, with array hybridization, gene expression measurement is limited by background at the low end and signal saturation at the high end, whereas RNAseq produces a more discrete sequencing read count, and thus can quantify expression across a greater dynamic range [16,17].

The IL-27pepL appears to modulate several changes that would be consistent with an anti-tumor effect, including upregulation of IFN, oxidative phosphorylation, JAK/STAT, and eIF2 signaling. Of these signaling changes predicted by IPA analyses, the one with highest significance and novelty was the eIF2α signaling upregulation. We validated this signaling change by assaying for eIF2α, p-eIF2α, and caspase-3 levels. We detected an increase in the phosphorylated form of eIF2α and in the cleaved caspase-3 fraction, indicating the eIF2α signaling pathway is upregulated in these prostate tumor cells following targeted IL-27 gene delivery, leading to apoptosis induction. This approach of targeting cytokines to enhance their activity against cancer cells is a novel approach to help augment IL-27′s bioactivity and efficacy against prostate tumors and could be extended to other conditions where it could help interfere with the eIF2α pathway and promote caspase-3 activation.

The regulators implicated in the coordination of the gene expression changes observed by RNAseq centered on the IFN/IRF/STAT1-2 pathways, as assessed by IPA and *Enrichr* dataset analyses. These related pathways have been shown to be linked to enhanced phosphorylation of eIF2α through IFN-induced phosphorylation of the eIF2α kinase 2 (PKR or EIF2AK2) through the activation of tyrosine kinase 2 (Tyk2) and Jak1 [18]. Despite the advances understanding the molecular processes driving the onset and progression of prostate and other cancers, only recently have defects of the cellular translation machinery begun to gain attention as an important cause of cancer development. In particular, the initiation step of translation has been established as having a key role in tumorigenesis. Induction of proapoptotic ER stress appears to be emerging as a key anticancer mechanism for certain agents targeting translation through inducing both the extrinsic and intrinsic apoptosis pathways.

Our results suggest that the targeted form of IL-27 may be utilized as a strategy impacting cell translation initiation through modulating the eIF2α pathway (ER stress leading to apoptosis) as a novel approach for treating prostate cancer. Although some have shown that prostate cancer cells can respond adaptively via eIF2α phosphorylation to reset global protein synthesis and promote aggressive tumor development [19], in other reports it appears that ER stress can lead a cell to either undergo apoptosis via upregulation of Fas cell surface death receptor (Fas) or to adapt via phosphorylation of eIF2α and upregulation of activating transcription factor 4 (ATF4), mechanisms that lead to sustained ER stress and result in cancer cell death via caspase-3 and others [20]. IL-27pepL appears to be able to activate an important checkpoint for maintaining epithelial cell homoeostasis, that is, an ER stress mechanism that contributes to apoptosis in prostate cancer cells. IL-27pepL expression during high proliferative TC2R conditions may promote impaired adaptive responses that render the cancer cells unable to cope with sustained ER stresses, leading to apoptosis. The IL-27pepL may be a way to target the adaptive brake for protein synthesis to selectively trigger cytotoxicity against aggressive prostate cancer cells. A potential additional advantage of the IL-27pepL therapy may be that an elevated level of p-eIF2α can accelerate the healing of bone wounds and enhance bone formation [21,22].

In conclusion, the present study performed an analysis of the RNAseq datasets for TC2R prostate adenocarcinoma modified to express IL-27ns or IL-27pepL and identified the top differentially expressed genes, pathways, as well as other biological functions for the novel IL-27pepL cytokine therapeutic. The IL-27pepL may be able to trigger p-eIF2α and target the ER stress response to tilt the balance towards apoptosis and for therapy, and may fall under the category of translation inhibitor that can induce proapoptotic ER stress [23] as an important direct antitumor mechanism on tumor cells.

## 4. Materials and Methods

### 4.1. Cell Culture

Mouse TRAMP–C2 cells were obtained from American Type Culture Collection (ATCC) and RM1 were a gift (S. Hayward) and both maintained in 1:1 Dulbecco’s modified essential medium and Ham’s F12 medium (DMEM:F12) (Mediatech, Manassas, VA, USA) with 10% fetal bovine serum (FBS) and 1× antibiotic-antimycotic (AA, ThermoFisher, Hanover Park, IL, USA). TRAMP-C2 cells were transduced with a lentivirus expressing activated H-ras^G12V^ at a multiplicity of infection of 1 (m.o.i. = 1) plus lentivirus transduction containing the mouse androgen receptor at m.o.i. = 1 each to generate the TC2R line [2], with the growth comparison between the parental TC2 and TC2R being described in [24]. TC2R cells were cultured in DMEM/F12 (ThermoFisher) with 10% FBS and 1× AA (ThermoFisher). RAW264.7 (murine monocytes) were obtained from ATCC (Manassas, VA, USA) and passaged by utilizing cell lifters. Cells were passaged by trypsinization (0.05% (v/v) trypsin, 0.53 mM ethylenediaminetetraacetic acid (EDTA) (ThermoFisher).

### 4.2. Vectors, Modeling, and Transfection Assays

The vectors utilized for gene delivery used a pcDNA3.1+ backbone, whereby the mouse hyper-IL-27 cDNA from pORF9-mEBI3/p28 (Invivogen, San Diego, CA, USA) was cloned by PCR amplification but with a 3′ insertion of a sequence encoding peptide linker (GGGGS) [25] plus the targeting peptide sequences (pepL: *LSLITRL* and as a non-specific (ns) control: Glu-Asp-Leu-Gly-Arg-Glu-Lys (*EDLGREK)*, previously shown to lack any specificity for IL-6/gp130 [26]). IL-27 cDNA-linker-peptide sequences were subcloned into pDrive (Promega, Madison, WI, USA), then excised and cloned into pcDNA3.1 using BamHI and NheI ends; empty vector control was pcDNA3.1 (pcDNA). Vectors were prepared for all experiments using Endofree kits (Qiagen, Valencia, CA, USA). For computational modeling, we utilized modeling by iTASSER based on the IL-6/gp130 protein databank (PDB) file 1p9m as a template to generate the mouse IL-27p28 structures. Then, we modeled the overlap between the p28 subunits that had been modified with peptides. The alignment was done using PyMol [27], predicting a similar structure for both p28 subunits and supporting the idea that the C-terminus-modified cytokine could remain active.

For RNAseq experiments, we delivered 1.5 ug of each plasmid vector to 5 × 10^5^ TC2R cancer cells seeded in a 6-well format the day prior (≈70–80% confluent the day of transfection). Transfection used plasmid DNA (1.5ug/well) in optiMEM media. Liposomes were prepared by adding Lipofectamine 2000 (2uL/well) to OptiMEM media and allowing equilibration for 5 min at room temperature (25 °C). Plasmid DNA/OptiMEM mixture was mixed with the liposome/OptiMEM mixture and incubated for 20 min at room temperature. The media was changed to OptiMEM (2.5ml/well) and pDNA/liposome complexes (200uL/well) added to each well, and transfection was allowed to proceed for 6h, after which the media was changed to 10% FBS DMEM/F12 (CellGro) with 1× antibiotic antimycotic (Gibco). Cells were collected 24h after the start of transfection in either RNAlater at 4 °C for the RNAseq studies or saved at −80 °C as a pellet for Western blot. Reporter firefly luciferase (Luc, Promega) assays used as constructs responsive to the active (phosphorylated) form of STAT1 (STAT1-Luc; LR0127, Panomics, Fremont, CA, USA) to transfect cells using Lipofectamine 2000 according to the manufacturer’s protocols for each cell type and cytokine stimulation, as described in [28]. Free peptides were synthesized and obtained with FITC conjugated to the N-terminus and an aminohexoic acid spacer (Ahx, Selleckchem (Houston, TX, USA). Cells were collected at 24 h of IL-27 (or control) stimulation, lysed in passive lysis buffer (Promega, Madison, WI, USA), and assayed in 96-well format using a Glomax luminometer with luciferin substrate (Promega). Flow cytometry for FITC detection utilized methods previously described [3,4].

### 4.3. RNAseq and Principal Component Analysis (PCA)

A cDNA library was constructed for three groups (pcDNA, pcDNA-IL-27ns, or pcDNA-IL-27pepL) with three samples per group using a TruSeq Stranded mRNA Library Prep kit (Illumina, San Diego, CA, USA). Sequencing was conducted with NextSeq500 (Illumina, San Diego, CA, USA). After quality control using FastQC (Babraham Bioinformatics, Cambridge, United Kingdom), sequenced libraries were mapped to the University of California Santa Cruz (UCSC) hg19 mouse genome. Principal component analysis (PCA) is a statistical procedure used to reduce the dimensions of a large dataset to help identify axes that best explain the variance of the data [29]. PCA was performed on the log2-transformed expression data. The gene and sample locations were plotted on the first and second principal axes.

### 4.4. Ingenuity Pathway Analyses (IPA)

RNAseq data were inputted into ingenuity pathway analysis (IPA, Qiagen, Germantown, MA, USA), as described in [30]. Briefly, by comparing the imported qPCR data with the Ingenuity Knowledge Base, a list of relevant networks, upstream regulators, and algorithmically generated mechanistic networks was obtained on the basis of their connectivity. Only genes with a *p*-value ≤0.05 were considered and both direct and indirect relationships were considered. Upstream regulator analysis was used to predict the upstream transcriptional regulators from the dataset on the basis of the literature and were compiled in the Ingenuity Knowledge Base. The analysis examined how many known targets of the upstream regulators were present in treated cell datasets and also the direction of change as compared to the control, with an activation z-score algorithm to make predictions. Downstream effect analysis was used to predict activation state if the direction of change was consistent with the activation state of a biological function. Top pathways and functions were scored by IPA and plotted as a heatmap, sorted by predicted activation and by number of molecules, and the top 5–10 pathways were depicted. IPA calculated a Benjamini–Hochberg (B-H) corrected *p*-value for upstream regulators and for causal networks, increasing the statistical stringency of these results in core analyses.

### 4.5. Western Blots

The experimental design followed the description in transfections, but the cells were collected as a pellet. For Western blotting, cells were lysed by a radio-immunoprecipitation assay buffer (Santa Cruz Biotech, Dallas, Texas, USA). Proteins were fractionated by 10%–15% SDS gels and electro-transferred to polyvinylidene difluoride membranes (Millipore, Billerica, MA, USA). Antibodies against caspase-3, cleaved caspase-3, eIF2α, p-eIF2α (Ser51), (Cell Signaling, Danvers, MA, USA), and β-actin (Sigma, St. Louis, MO, USA) were utilized. Densitometry analyses utilized National Institutes of Health (NIH) ImageJ software (available online: http://imagej.nih.gov/ij/, accessed on 5 February 2020), analyzing band intensity quantified from the 16-bit digital image by using the gel analysis plug-in.

### 4.6. Statistical Analyses

Assays were performed in triplicate and values provided as mean ± standard error of the mean (SEM) or 95% confidence interval. Comparisons were performed using unpaired *t*-tests or one-way analysis of variance analysis using the Bonferroni *t*-test and *p* < 0.05 considered as indicating a significant difference.

## Abbreviations

eIF2α	eukaryotic translation initiation factor 2 alpha
ER	endoplasmic reticulum
DOAJ	directory of open access journals
IL-27	interleukin-27
IL-6-Rα	interleukin-6 receptor alpha
IPA	ingenuity pathway analyses
MDPI	Multidisciplinary Digital Publishing Institute
PCA	principal component analysis
TC2R	TRAMPC2-Ras murine prostate adenocarcinoma cells
RM1	Ras/Myc murine prostate adenocarcinoma cells
RNAseq	RNA sequencing
